# Supplementary material for: WordCommentsAnalyzer: A windows software tool for qualitative research
Source: F1000Res. 2018 Sep 4;7:536. Originally published 2018 May 3. [Version 2] doi: 10.12688/f1000research.14819.2 (PMC6137411; doi:10.12688/f1000research.14819.2)
Supplement: Supplementary file 3 [file f1000research-7-17614-s0002.tgz › b2a79496-4ada-47c4-9682-a99ce867836d.zip!/Eight Iranian Health Journals/Journal of Research in Rehabilitation Sciences _13.docx]

© Journal of Research in Rehabilitation Sciences . This work is licensed under CC BY-NC 4.0 (https://creativecommons.org/licenses/by-nc/4.0/).

Volume & Issue: دوره 11، شماره 4: 1394

Title: بررسی رابطه ترس از زمین خوردن با میزان تحصیلات و وضعیت زندگی سالمندان

http://jrrs.mui.ac.ir/index.php/jrrs/article/view/2064

مقدمه: هدف از انجام پژوهش حاضر، بررسی رابطه ترس از زمین خوردن با میزان تحصیلات و وضعیت زندگی سالمندان ایرانی بود. مواد و روش‌ها: این مطالعه از نوع توصیفی- تحلیلی بود. 191 سالمند مقیم جامعه و آسایشگاه سالمندان به شیوه نمونه‌گیری غیر احتمالی ساده برای شرکت در این طرح انتخاب شدند. میزان ترس از زمین خوردن، میزان تحصیلات و شیوه زندگی بر اساس پرسش‌نامه‌های چند گزینه‌ای مشخص گردید و رابطه بین آن‌ها بررسی شد. روش‌‌های آماری شامل آزمون‌ Kruskal-Wallis و ضریب همبستگی Spearman بود. یافته‌ها: بر اساس نتایج آزمون Kruskal-Wallis، ترس از زمین خوردن بین افراد با شیوه‌های مختلف زندگی، تفاوتی مشاهده نگردید (25/0 = P). با محاسبه ضریب همبستگی Spearman نیز رابطه‌ای بین میزان ترس از زمین خوردن با سطح تحصیلات دیده نشد (07/0 = R، 32/0 = P). نتیجه‎گیری: یافته‌های این پژوهش نشان ‌داد که ارتباطی بین ترس از زمین خوردن با میزان تحصیلات و همچنین، وضعیت زندگی افراد سالمند وجود نداشته است.

Title: غربالگری عملکرد حرکتی دانشجویان آموزشکده فنی شهرکرد جهت پیشگیری از آسیب‌های ورزشی

http://jrrs.mui.ac.ir/index.php/jrrs/article/view/2391

مقدمه: پیشگیری و کاهش خطر آسیب‌دیدگی، نسبت به درمان آسیب اهمیت بیشتری دارد. یکی از اهداف مهم علوم ورزشی، پیشگیری از آسیب است که با غربالگری پیش از شرکت در فعالیت ورزشی، می‌توان به بخش بزرگی از این مهم دست یافت. هدف از انجام پژوهش حاضر، غربالگری عملکرد حرکتی دانشجویان جهت پیشگیری از آسیب‌های ورزشی بود. مواد و روش‌ها: نمونه آماری پژوهش حاضر، شامل 180 دانشجوی پسر (سن 0/1 ± 1/18 سال، قد 56/0 ± 76/1 متر و وزن 3/13 ± 2/65 کیلوگرم) بود. از آزمون‌های غربالگری عملکرد حرکتی (Functional movement screening یا ‌FMS) شامل دیپ اسکات، گام برداشتن از روی مانع، لانچ، تحرک‌پذیری شانه، بالا آوردن فعال پا، شنای پایداری تنه و ثبات چرخشی استفاده شد. به منظور تجزیه و تحلیل داده‌ها و بررسی ارتباط بین سطح عملکردی آزمودنی‌ها (خوب، متوسط و ضعیف) با نوع تست عملکردی، از آزمون استنباطی 2χ استفاده گردید (01/0 > P). یافته‌ها: در پژوهش حاضر، 66 نفر (7/36 درصد) نمرات زیر 14 کسب کردند. بیشترین نقص حرکتی مربوط به الگوهای ثبات چرخشی (7/26 درصد)، کشش فعال بالا آوردن پا (6/25 درصد)، تحرک شانه (2/17 درصد) و لانچ (6/15 درصد) بود (010/0 > P). نتیجه‎گیری: با توجه به یافته‌های پژوهش، تعداد زیادی از دانشجویان در اجرای الگوها ضعف داشتند و در معرض آسیب و مشکلات عملکردی بودند. بنابراین، از آزمون‌های غربالگری عملکردی، می‌توان به منظور غربالگری پیش از شرکت در فعالیت با هدف، مشخص کردن افراد مستعد آسیب و انجام اقدامات پیشگیرانه استفاده کرد.

Title: بررسي تأثير ماساژ افلوراژ بر ميزان تحمل و حداکثر قدرت مشت کردن، پس از خستگي در زنان جوان سالم

http://jrrs.mui.ac.ir/index.php/jrrs/article/view/2469

مقدمه: دست یکی از مهم‌ترین اندام‌های انسان است که در بسیاری از فعالیت‌ها مانند عمل گرفتن، از آن استفاده می‌شود. از سوی دیگر، انجام فعالیت طولاني مدت عضلات منجر به خستگی می‌گردد. یکی از راه‌های کاهش این خستگی، انجام ماساژ است. مطالعات انجام شده در خصوص تأثیر ماساژ بر قدرت و تحمل عضلانی محدود می‌باشد. علاوه بر این، همخوانی در نتایج مطالعات مشابه در دسترس، وجود ندارد. بنابراین، هدف از انجام پژوهش حاضر، استفاده از یک روش ساده ماساژ و بررسی تأثیر آن بر قدرت و تحمل عضلانی به دنبال خستگی بود. مواد و روش‌ها: 49 نفر از دانشجویان دختر 18 تا 30 ساله دانشکده علوم توان‌بخشی شیراز، به روش تصادفي برای شرکت در پژوهش انتخاب شدند. آزمون‌ها شامل اندازه‌گیری حداکثر قدرت و زمان تحمل مشت کردن بود. تأثیر ماساژ و استراحت به تنهایی، بر این دو متغیر در 2 جلسه مجزا بررسی گردید. برای متغیر حداکثر قدرت عضلانی، از آزمون Paired t و جهت متغیر زمان تحمل عضلانی از معادل ناپارامتری آن، یعنی آزمون Wilcoxon استفاده گردید. در نهایت، داده‌ها در نرم‌افزار SPSS مورد تجزیه و تحلیل قرار گرفت. یافته‌ها: در پژوهش حاضر، قدرت مشت کردن پس از خستگی کاهش یافت. انجام ماساژ افلوراژ پس از خستگی، به طور معنی‌داری نسبت به گروه شاهد باعث افزایش قدرت مشت کردن گردید (050/0 > P). نتیجه‎گیری: انجام ماساژ افلوراژ، منجر به بهبود قدرت مشت کردن پس از خستگی ‌گردید؛ در حالی که بر میزان تحمل مشت کردن اثری نداشت.

Title: کارامدی راهبرد مرور ذهنی بر حافظه کوتاه‌مدت دیداری، در کودکان مبتلا به نشانگان داون

http://jrrs.mui.ac.ir/index.php/jrrs/article/view/2111

مقدمه: نشانگان داون (Down syndrome)، شایع‌ترین علت ژنتیکی اختلالات یادگیری (به طور متوسط ۲۲ درصد) می‌باشد. حافظه کاری که وظیفه ذخیره اطلاعات را به عهده دارد و در یادگیری اهمیت زیادی دارد، در این افراد دچار اختلال می‌شود. هدف از انجام پژوهش حاضر، بررسی تأثیر راهبرد مرور ذهنی (Rehearsal strategy) بر حافظه کوتاه‌مدت دیداری (Visual short term memory) کودکان آموزش‌پذیر ۵ تا ۸ ساله مبتلا به نشانگان داون بود. مواد و روش‌ها: در این تحقیق شبه آزمایشی، پس از اخذ رضایت والدین، ۲۴ نفر از کودکان مبتلا به نشانگان داون که عضو کانون خیریه سندرم داون بودند، به روش در دسترس انتخاب شدند. سپس، به وسیله ماتریس‌های پیش‌رونده هوش Raven مورد بررسی قرار گرفتند. کودکان به طور تصادفی به دو گروه آزمایش و شاهد تقسیم شدند. گروه آزمایش در ۸ جلسه تحت آموزش راهبرد مرور ذهنی قرار گرفتند. ۲۴ کودک شرکت کننده، قبل و بعد از مداخله آموزشی توسط آزمون توالی دیداری حافظه ارزیابی شدند. اطلاعات به دست آمده، بر اساس آزمون MANCOVA تجزیه و تحلیل شد. یافته‌ها: راهبرد مرور ذهنی باعث افزایش فراخنای عدد (Digit span) و کلمه (Word span) و تقویت حافظه کوتاه‌مدت دیداری (001/0 < P)، در کودکان گروه آزمایش شد. نتیجه‎گیری: یافته‌های پژوهش حاضر نشان داد که راهبرد مرور ذهنی یکی از روش‌های اصلی است که می‌توان در جهت ارتقای حافظه کوتاه‌مدت دیداری کودکان مبتلا به نشانگان داون به کار برد.

Title: بررسی روایی همزمان مقیاس خودسنجی کاری کودک، در کودکان با نقص توجه و بیش‌فعالی

http://jrrs.mui.ac.ir/index.php/jrrs/article/view/2412

مقدمه: استقلال در فعالیت‌های روزمره زندگی، جزء اولین موفقیت‌های دوران کودکی است که به ارتقای حس استقلال و اجتماعی شدن کودکان كمک می‌نمايد. پژوهش حاضر با استفاده از پرسش‌نامه‌های‌ خودسنجی کاری کودک و استاندارد کیفیت زندگی کودکان (که به ارزیابی فعالیت‌های روزمره زندگی اختصاص دارد) صورت گرفت. هدف از انجام این پژوهش، بررسی روایی همزمان مقیاس خودسنجی کاری کودک، در کودکان دارای اختلال نقص توجه و بیش‌فعالی (ADHD یا Attention deficit hyperactivity disorder) بود. مواد و روش‌ها: پژوهش حاضر از نوع همبستگی بود. جهت بررسی روایی هم‌زمان، هر دو پرسش‌نامه توسط 128 کودک با ADHD تکمیل شد. تحلیل نتایج و گزارش ضرایب همبستگی در نرم‌افزار SPSS مورد تجزیه و تحلیل قرار گرفت. یافته‌ها: بیشترین ضریب همبستگی بین مقیاس‌ها به کفایت و کیفیت زندگی کل کودک تعلق داشت (56/0- = r). کمترین ضریب نیز متعلق به نمره کفایت با عملکرد اجتماعی برابر با 44/0- = r بود (01/0 > P). بیشترین و کمترین ضرایب همبستگی در مقیاس ارزش به ترتیب به کیفیت زندگی کل کودک (46/0- = r) و عملکرد احساسی (34/0- = r) اختصاص داشت (01/0 > P). نتیجه‌گیری: مقیاس خودسنجی کاری کودک با پرسش‌نامه استاندارد کیفیت زندگی کودکان، روایی همزمان قابل قبولی را نشان داد که می‌تواند به عنوان ابزاری برای سنجش کودکان با ADHD به کار رود.

Title: ویژگی‌های کینماتیکی و فضایی- زمانی راه رفتن نابینایان

http://jrrs.mui.ac.ir/index.php/jrrs/article/view/2415

مقدمه: درون‌دادهای بینایی در فرایند پاسخ حرکتی سیستم عصبی- عضلانی مرکزی، در وظایف مختلف حرکتی نقش مهمی دارند. شناسایی اثر فقدان اطلاعات بینایی در افراد نابینا هنگام راه رفتن، می‌تواند در طراحی توان‌بخشی و اصلاح الگوی حرکتی آن‌ها مؤثر باشد. هدف از انجام پژوهش حاضر، مقایسه ویژگی‌های فضایی- زمانی، تقارن گام‌برداری افراد سالم و نابینا و شناسایی اثر بستن چشم در راه رفتن افراد سالم بود. مواد و روش‌ها: تعداد 10 نفر نابینا و 10 نفر سالم با سن، قد و وزن مشابه در این پژوهش مورد مطالعه قرار گرفتند. متغیرهای کینماتیکی فضایی- زمانی راه رفتن آزمودنی‌ها در شرایط راه رفتن افراد نابینا بدون عصا و راه رفتن افراد سالم با چشم باز و چشم بسته محاسبه گردید. تفاوت بین گروه‌ها و دو موقعیت گام‌برداری با استفاده از روش Repeated measures و با سطح معنی‌داری 05/0 P < شناسایی شد. یافته‌ها: طول گام و طول قدم و سرعت گام‌برداری در افراد نابینا کمتر از گروه سالم با چشم باز بود (050/0 >P ). همچنین، طول گام و طول قدم و نیز زمان گام افراد سالم در شرایط چشم بسته، بزرگ‌تر از این مقادیر در افراد نابینا بود. در گروه سالم متغیرهای زمان گام و زمان قدم، زمان حمایت تک اتکایی و زمان نوسان با بستن چشم افزایش و Cadence کاهش یافت. همچنین، شاخص عدم تقارن در افراد سالم با چشم بسته در متغیرهای مدت زمان اتکای یک پا، زمان نوسان و زمان جدا شدن پنجه از زمین، تقارن بیشتری در مقایسه با افراد نابینا داشت.   نتیجه‌گیری: نابینایی با کاهش سرعت راه رفتن، طول گام و قدم همراه است. بستن چشم در افراد سالم موجب افزایش زمان گام، زمان حمایت تک اتکایی، زمان نوسان و کاهش Cadence می‌گردد. متغیرهای طول گام و قدم و زمان گام‌برداری در افراد سالم با چشم بسته بزرگ‌تر از این مقادیر در نابینایان بود. به نظر می‌رسد نقش گیرنده‌های حسی- عمقی در نابینایان بیشتر از افراد سالم است.

Title: مروری بر تأثیرات نوروپاتولوژیک هسته‌های قاعده‌ای در بروز اختلال لکنت

http://jrrs.mui.ac.ir/index.php/jrrs/article/view/2411

هسته‌های قاعده‌ای، گروهی از ساختارهای زیرقشری هستند که به صورت یک واحد عملکردی پیوسته عمل می‌کنند. این هسته‌ها در قاعده‌ی مغز پیشین قرار گرفته‌اند و دارای ارتباطات گسترده‌ای با قشر مغز و تالاموس می‌باشند. هسته‌های قاعده‌ای با عملکردهای مختلفی مانند کنترل حرکتی ارادی، یادگیری، حرکات چشم، شناخت و عاطفه مرتبط هستند و از چهار چرخه حرکتی، شناختی، سیستم لیمبیک و حرکات چشم تشکیل شده‌اند. چرخه‌ حرکتی دارای مسیری قشری- قاعده‌ای- قشری است. لکنت شامل اختلال در تولید گفتار همراه با قفل کردن، کشیده‌گویی مکرر یا مکث صوتی است. علت اصلی لکنت مشخص نیست. برخی از اختلالات گفتاری مانند لکنت می‌تواند نتیجه‌ اختلال در چرخه‌ بین هسته‌‌های قاعده‌ای و نواحی حرکتی زبانی قشر مغز باشد. هسته‌های قاعده‌ای به سبب ارتباط با قشر مغز، به خصوص ناحیه‌ بروکا و قشر حرکتی گفتار، بسیاری از مشخصه‌های گفتاری را متأثر می‌کنند. در این مقاله‌ مروری، ارتباط‌های احتمالی بین لکنت و هسته‌های قاعده‌ای مورد بحث قرار گرفت.

Title: بررسی الگوی حرکات تنه در کودکان مبتلا به فلج مغزی: یک مرور نظام‌مند

http://jrrs.mui.ac.ir/index.php/jrrs/article/view/2394

مقدمه: فلج مغزی یک ناهنجاری غیر پیشرونده است که منجر به نقایص جسمی و عملکردی می‌گردد. بیش از نیمی از کودکان مبتلا به فلج مغزی به راه رفتن مستقل دست می‌یابند، اما الگوی راه رفتن در اکثر این کودکان ناهنجار است. با توجه به این که تنه در قسمت مرکزی بدن واقع شده است، در کنترل وضعیت و سازماندهی واکنش‌های تعادلی نقش تعیین کننده‌ای دارد. هدف از انجام این مرور نظام‌مند، بررسی الگوی حرکات تنه در کودکان مبتلا به فلج مغزی، متناسب با نوع و شدت فلج مغزی و چگونگی تأثیر حرکات تنه بر کنترل حرکات بود. مواد و روش‌ها: کلید واژه‌های «Gait و Trunk» در ترکیب با «Cerebral palsy» در پایگاه‌های اطلاعاتی PubMed، Google Scholar، Scopus،ProQuest  و Web of Science جستجو گردید. تاریخ جستجو از زمان آغاز به کار این پایگاه‌ها تا مارس سال 2015 در نظر گرفته شد. فرایند جستجو به طور مستقل توسط دو نفر از نویسندگان صورت گرفت. برای ارزیابی کیفیت مقاله‌ها از مقیاس PEDro و برای ارزیابی سطح شواهد از مقیاس CEBM ‎levels of evidence scale (Centre for evidence-based medicine) استفاده شد. پس از فرایند جستجو و متناسب با معیار ورود و خروج، 5 مقاله وارد مطالعه شد. یافته‌ها: مقاله‌ها کیفیت متوسط تا پایینی داشتند. آن‌ها افزایش حرکات تنه را متناسب با افزایش شدت فلج مغزی گزارش نمودند. حرکات ایجاد شده، می‌تواند به عنوان حرکات جبرانی و یا به عنوان نقص کنترل حرکتی تنه در نظر گرفته شود. نتیجه‎گیری: دامنه حرکات تنه متناسب با شدت فلج مغزی افزایش می‌یابد. با این حال، جهت آگاهی از چگونگی الگوی حرکات تنه متناسب با نوع و شدت فلج مغزی، باید مطالعات بیشتری صورت گیرد. همچنین، هنوز این سؤال باقی است که آیا حرکات تنه نقش جبرانی دارند و یا باید به عنوان یک نقص تلقی گردند.
